# Work-Related Musculoskeletal Symptoms among Building Construction Workers in Riyadh, Saudi Arabia

**DOI:** 10.12669/pjms.296.4052

**Published:** 2013

**Authors:** Sultan Ayoub Meo, Zaid Fahad Alsaaran, Moayad Khalid Alshehri, Mohammed Azam Khashougji, Abdul Aziz Zayed Almeterk, Saif Fraj Almutairi, Saad Fahad Alsaeed

**Affiliations:** 1 Sultan Ayoub Meo, MBBS, PhD, FRCP, Professor, Department of Physiology, College of Medicine, King Saud University, Riyadh, Saudi Arabia.; 2 Zaid Fahad Alsaaran, MBBS St, College of Medicine, King Saud University, Riyadh, Saudi Arabia.; 3 Moayad Khalid Alshehri, MBBS St, College of Medicine, King Saud University, Riyadh, Saudi Arabia.; 4 Mohammed Azam Khashougji, MBBS St, College of Medicine, King Saud University, Riyadh, Saudi Arabia.; 5 Abdul Aziz Zayed Almeterk, MBBS St, College of Medicine, King Saud University, Riyadh, Saudi Arabia.; 6 Saif Fraj Almutairi, MBBS St, College of Medicine, King Saud University, Riyadh, Saudi Arabia.; 7 Saad Fahad Alsaeed, MBBS St, College of Medicine, King Saud University, Riyadh, Saudi Arabia.

**Keywords:** Building construction, Musculoskeletal symptoms, Occupational hazards

## Abstract

***Objectives: ***To investigate the work-related musculoskeletal symptoms among building construction workers.

***Methods: ***Total 389 apparently healthy, male volunteers were selected with mean age 34.56±8.33 years and a mean working duration in building construction as 5.76±2.68 years. Musculoskeletal complaints were recorded through a detailed clinical interview and comprehensive questionnaire.

***Results: ***Substantial number of building construction workers developed musculoskeletal symptoms including neck pain 29 (7.5%), shoulder pain 41(10.5%), upper back pain 24(6.2%), lower back pain 64 (16.5%), legs pain 93 (23.9%), feet pain 52 (13.4%), head heaviness 44 (11.3%) and whole body fatigue 78 (20.1%). These complaints were significantly associated with long-term duration-response in building construction industry. Furthermore, cigarette smokers had little higher percentage of musculoskeletal complaints compared to non-smoker companions.

***Conclusions: ***Building construction occupation is a prolific source of musculoskeletal ailments and complaints were significantly increased with long-term working duration in building construction industry.

## INTRODUCTION

The worldwide community, especially the people in developing countries is facing increasing risks of various systemic ailments due to working exposure in different occupational and industrial sectors.^1^ Building construction is a large, dynamic and complex industrial sector, creating employment for millions of people worldwide on its workforce. This industry has a pivotal role in the construction of the infrastructure. The building construction workers are involved in different trades like mason, cement mixer, plumber, electrician, carpenter, and crane operator etc.^2^

Building construction workers face a wide range of health hazards in their work sites and are victims of occupational health hazards. These may include exposure to dust, fumes, holding weighty materials and heavy physical work in awkward working postures in the construction industry which can cause various musculoskeletal disorders. Moreover, exposure to tough environmental conditions affects the worker's health and causes various physiological, physical and psychosocial strain and workers become victims of occupation related musculoskeletal disorders^3^ and accidents.^2^

Furthermore, these workers have fatal injuries on-the-job and high numbers of non-fatal occupational injuries, illnesses and workplace mortality.^4^ The worldwide industrial labor community especially the people in developing countries is facing increasing risks of various ailments^1^ including musculoskeletal disorders.^5^ The health risks posed by industry are influenced by type, nature of job and duration of working in various industrial sectors.^6^^,^^7^ The musculoskeletal disorders are most common occupational related health problems and are an important cause of functional impairments and disability among building construction workers.^8^

This study aimed to investigate the musculoskeletal symptoms among workers working in building construction industry and also provide information to workers about the working hazards in building construction industries.

## METHODS

The present descriptive study was conducted in the Department of Physiology, College of Medicine, King Saud University, Riyadh, Saudi Arabia during the period Oct 2012-March 2013. 540 building construction workers were interviewed; a comprehensive clinical history was taken. The trades of the subjects were as follows: cement mixer 104 (26.7%); concrete workers 42 (10.8%); steel fixer 38 (9.8%); crane operator 15 (3.9%); electrician 48 (12.3%); plumber 37 (9.5%); masons 18 (4.6%); supervisor 50 (12.9%); and general supporters 37 (9.5%). Among them, 300 (77.13%) were non-smokers and 89 (22.13%) were smokers (mean average smoking per day was 4.48±1.49). Building construction workers were further divided into three groups based on the duration of exposure less than 3 (131, 33.67%); 3-6 (111, 28.53%) and greater than 6 years (147, 37.78%). These workers worked for at least 8-10 hours a day for six days per week.

All the subjects voluntarily participated in the study and signed the consent form. The Institutional Ethical Committee approved the study.Subjects with known history of gross anemia, diabetes mellitus, pulmonary tuberculosis, malignancy, rheumatoid arthritis, joint deformities were excluded from the study.

A well-structured English language questionnaire was developed and was also translated into Arabic, Urdu and Hindi languages. The questionnaire consisted the anthropometric variables, cigarette smoking, occupation conditions, working exposure, any known history of present and past illness, exposure conditions during the working hours, and questions about musculoskeletal problems. The co-authors informed the participants about the objectives of the study. Subjects were questioned about complaints of the musculoskeletal system. The complaints score reflected the presence or absence of the symptoms. 540 questionnaires were distributed among them 389 (72.03%) participants completed and 151 (27.96%) either did not fully completed the questionnaire or were excluded from the study as per exclusion criteria. Finally, we included 389 apparently healthy, volunteer males with mean age 34.56±8.33 years (mean SD; range 22-54) years who had a working duration in building construction as 5.76±2.68 years (mean SD; range 1-12 years).

 ***Statistical analysis: ***The data were entered into the computer; Statistical Package for the Social Sciences (SPSS) software version 19.0 was used. Musculoskeletal findings were recorded in number and percentage (%). Odd Ratio was computed for musculoskeletal complaints with 95% confidence interval. Chi square test and Chi square linear trend were used to compare the musculoskeletal symptoms among subjects. Spearman correlation coefficient was calculated to find out the relation between the age, duration of working in construction industry with musculoskeletal complaints. The level of significance was achieved at p-value less than 0.05.

## RESULTS

**Fig.1:**
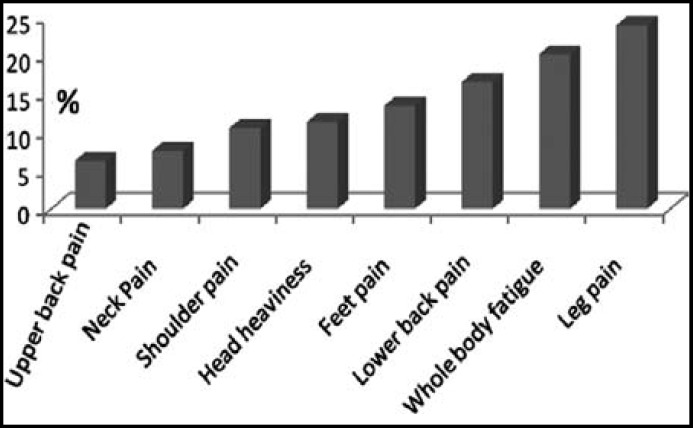
The occurrence of musculoskeletal symptoms in building construction workers

Three hundred eighty nine(389) apparently healthy subjects with mean age 34.56±8.33 ±±2.68 years ±1-12 years) participated in the study.A large proportion of the subjects had complaints of neck pain 29 (7.5%), shoulder pain 41(10.5%), upper back pain 24(6.2%), lower back pain 64 (16.5 %), legs pain 93 (23.9%), feet pain 52 (13.4%), head heaviness 44 (11.3%), and whole body fatigue 78 (20.1%) (Table-I, Fig.1).

Table-II shows the musculoskeletal complaints based on duration of years in building construction industry. Workers with more than 6 years had significantly higher musculoskeletal symptoms compared to those companions who worked for less than 3 years or 3-6 years.

The musculoskeletal complaints were associated with duration of work in building construction industry: neck pain (rs=-0.15, p=0.002); shoulder pain (rs=-0.104,p=0.02); upper back pain (rs=-0.065, p=0.10); lower back pain (rs=-0.135, p=0.004); leg pain (rs=-0.223, p=0.0001); feet pain (rs=-0.161, p=0.001); head heaviness (rs=-0.088, p=0.042); and whole body fatigue (rs=-0.228, p=0.0001). Moreover, the results also show a significant correlation between age and few musculoskeletal complaints including upper back pain (rs=-0.116, p=0.011); lower back pain (rs=-0.093, p=0.034) however, there was no correlation between the age and other musculoskeletal problems.(Table-III)

**Fig.2:**
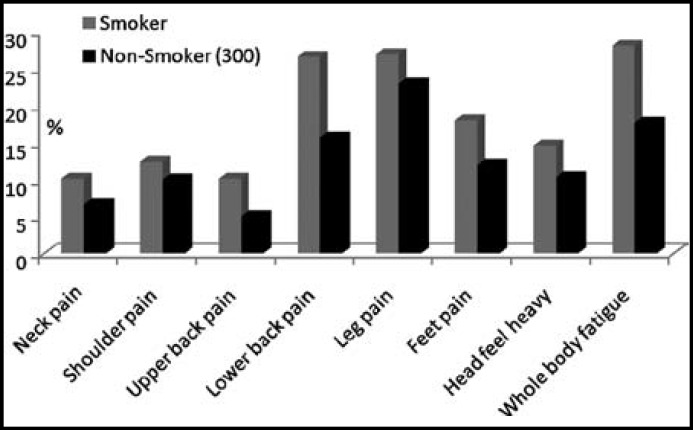
Comparison of musculoskeletal symptoms between smoker and non-smoker building construction workers

Table-IV shows that cigarette smokers had little higher percentage of musculoskeletal complaints but these complaints did not achieve a level of significance except for the whole body pain between the smokers and non-smokers (p=0.03) (Fig.2).

**Table-I:** Musculoskeletal symptoms in building construction workers (n=389).

*Musculoskeletal Symptoms*	*Total number and percentage*	*95% CI*
Neck Pain (n=29)	29 (7.5%)	5.1 -10.0
Shoulder pain (n=41)	41 (10.5%)	7.7-14.1
Upper back pain (n=24)	24 (6.2%)	4.1-8.5
Lower back pain (n=64)	64 (16.5%)	12.3-20.1
Legs pain (n=93)	93 (23.9%)	19.7-27.5
Feet pain (n=52)	52 (13.4%)	10-16.5
Head Heaviness (n=44)	44 (11.3%)	8.2-14.4
Whole body fatigue (n=78)	78 (20.1%)	16.2-24.2

Number and percentages, 95% CI = 95% Confidence interval

**Table-II:** Musculoskeletal symptoms in building construction workers based on the duration of working years in building construction [Musculoskeletal symptoms presented in yes or no format] (n=389).

*Musculoskeletal Symptoms*	*Duration*			*P-value*
	*Less than 3 year(n=131)*	*3-6 years* *(n=111)*	*More than 6 years* *(n=147)*	
Neck pain (n=29)	5 (3.8 %)	5 (4.5 %)	19 (12.9 %)	0.003
Shoulder pain (n=41)	11 (8.4 %)	7 (6.3 %)	23 (15.6 %)	0.044
Upper back pain (n=24)	7 (5.3 %)	4 (3.6 %)	13 (8.8 %)	0.212
Lower back pain (n=64)	14 (10.7 %)	17 (15.3 %)	33 (22.4 %)	0.008
Legs pain (n=93)	17(13.0 %)	24 (21.6 %)	52(35.4 %)	0.0001
Feet pain (n=52)	11 (8.4 %)	10 (9.0 %)	31 (21.1 %)	0.002
Head feel heavy (n=44)	9 (6.9 %)	15 (13.5 %)	20 (13.6 %)	0.082
Whole body fatigue (n=78)	15 (11.5 %)	15 (13.5 %)	48 (32.7 %)	0.0001

* Statistical significant difference since p-value < 0.05, by Chi-square linear trend test

**Table-III:** Spearmen correlation coefficient between age and duration of working years in building construction industry with musculoskeletal symptoms (n=389).

*Musculoskeletal Symptoms*	*Age *	*Duration*
Neck pain	rs =- 0.064 p=0.105	rs =- 0.15 p=0.002
Shoulder pain	rs =-0.051 p =0.158	rs =-0.104 p =0.02
Upper back pain	rs =-0.116 p =0.011	rs =-0.065 p =0.102
Lower back pain	rs =- 0.093 p=0.034	rs =- 0.135 p=0.004
Legs pain	rs =0.037 p =0.235	rs =-0.223 p =0.0001
Feet pain	rs =-0.039 p =0.220	rs =-0.161 p =0.001
Head feel heavy	rs =-0.025 p =0.313	rs =-0.088 p =0.042
Whole body fatigue	rs = 0.077 p=0.066	rs = -0.228 p=0.0001

rs= Spearmen correlation coefficient, p=p-value

**Table-IV:** Comparison of musculoskeletal symptoms between smoker and non-smoker building construction workers(n=389).

*Musculoskeletal Symptoms*	*Smoker* *(89)*	*Non-Smoker (300)*	*Odds ratio*	*95% Confidence intervals*	*P-value*
Neck pain (29)	9 (10.1 %)	20 (6.7 %)	1.575	0.69 – 3.594	0.277
Shoulder pain (41)	11 (12.4 %)	30 (10.0 %)	1.269	0.608 – 2.648	0.524
Upper back pain (24)	9 (10.1 %)	15 (5.0 %)	2.138	0.902 – 5.065	0.078
Lower back pain (64)	17(26.6 %)	47 (15.66 %)	1.27	0.688 –2.347	0.443
Leg pain (93)	24 (26.96 %)	69 (23 %)	1.236	0.72 – 2.12	0.441
Feet pain (52)	16 (18.0 %)	36 (12.0 %)	1.607	0.845 – 3.059	0.146
Head feel heavy (44)	13 (14.6 %)	31 (10.3 %)	1.484	0.740 – 2.977	0.264
Whole body fatigue (78)	25 (28.1 %)	53 (17.7 %)	1.820	1.051 – 3.153	0.031

Number and percentages, Odd ratio, 95% CI = 95% Confidence interval

## DISCUSSION

Work related occupational exposure to building construction industry and its effects on human health is a leading health problem. We found that high number of the building construction workers developed musculoskeletal problems including neck pain 7.5%, shoulder pain 10.5%, upper back pain 6.2%, lower back pain 16.5%, leg pain 23.9%, feet pain 13.4%, head heaviness 11.3%, and whole body fatigue 20.1%. These complaints were associated with long term **work** duration in building construction industry.

Kilbom *et al.*^9^ reported that increased frequency in repetitive work can cause discomfort and pain in the neck, shoulders, and upper extremities. It seems to attribute pain reporting to mechanical tissue overload related to repetitive movements, force requirements, and awkward postures. It is also possible that repetitive work could lead to stress symptoms and musculoskeletal pain.^10^

Boschman *et al.*^8^ reported that subjects working in building construction industry had complaints of the back, knee and shoulder / upper arm pain and most of the workers reported that their complaints are work-related. Complaints of the back and elbow were the most often reported among the workers during work, whereas lower arm/wrist and upper leg complaints were the most often reported among the supervisors.

In parallel to our findings, Rosecrance *et al.*^11^ conducted a study in building construction workers and demonstrated that the musculoskeletal symptoms were common in neck 24.7%, shoulders 18.9%; upper back 28.7%; elbows 8.0%; low back 45.0%; wrist / hands 29.6%; hip / thighs 4.7%; knee 10.9% and ankles / feet 10.7 %. The authors demonstrated that bending or twisting the neck, back in awkward positions, static positions and materials handling were reported as the most causative job factors. Congruently, Battevi and Vitelli^12^ reported a significant increase in work-related musculoskeletal disorders. They found that, a group of construction workers exhibited high prevalence of upper limb disorders. Bye^13^ conducted a study on the employees in construction industry in order to determine the prevalence of musculoskeletal strain. The author found that, 40.3% of the employees had pain at the time of questioning and the percentage of the complaints increases gradually with age. In the present study, we found that building construction workers had higher upper back pain and lower back pain with increasing age (Table-III).

Hanklang *et al.*^14^ determine the prevalence of musculoskeletal symptoms among building construction workers. The findings revealed that 57.7% of workers reported musculoskeletal symptoms with low back and shoulders as the most common body parts affected (46.0%). Multiple logistic regression analysis indicated that prolonged work duration was significantly associated with musculoskeletal disorders. Similarly in the present study, we found a duration-response relationship with longer exposures leading to increased musculoskeletal complaints.

Guo *et al*.^15^ found that building construction workers are at high-risk for musculoskeletal disorders among the working population. Neck and shoulder disorders affect 26.3% of male construction workers. Ashish *et al.*^16^ reported that building construction workers spend ample time in lifting, holding, carrying, pulling or pushing loads of material. They reported lifting and holding heavy weights as a source of musculoskeletal disorders involving neck among construction workers. Similarly, based on the preset study results we believe that, work-related musculoskeletal symptoms in building construction workers occur when mechanical workload is higher than physical capacity of human body and this can cause repeated trauma in the musculoskeletal system.

Gallagher^17^ also found that building construction workers had higher risk of musculoskeletal complaints. Yu-Sheng *et al.*^18^ conducted a study among building construction workers and their result showed that 76.2% of the workers reported musculoskeletal complaints. Shoulder symptoms were reported as the most prevalent work-related symptoms (47.6%), neck pain was the second (43.8%), and low back pain was the third (38.1%). Similarly in the present study we found that the musculoskeletal symptoms were common in neck, shoulders, upper back, low back, legs and feet. The findings of the present study are in agreement with the study of Yu-Sheng et al.^18^ however, in the present study, the percentages of symptoms were less compared to Yu-Sheng *et al*.^18^ The mechanism associated with musculoskeletal symptoms in building construction workers is mainly due to repetitively lifting and carrying heavy objects and also working in tough environment. The sustained activities can be linked to repetitive work, loading of tendons, rupture of the muscle fibers along with muscle disorders.^19^ Moreover, manual handling of building construction materials in different awkward postures increases the risk of musculoskeletal disorders.^20^

## CONCLUSION

The present study adds evidence to the existing literature that a large proportion of labor force working in building construction had higher complaints of neck pain, shoulder pain, upper back pain, lower back pain, legs pain, feet pain, head heaviness and whole body fatigue. Moreover, construction workers working for more than 6 years had higher musculoskeletal symptoms. Cigarette smokers had little higher percentage of musculoskeletal complaints compared to non-smoker companions. The results indicate that building construction job is a prolific source of musculoskeletal ailments and complaints significantly increased with long-term work duration in building construction industry. It is advisable that health risk should be reduced by the mutual collaboration between health officials, building construction workers and their management to adopt appropriate protective measures and to avoid lifting heavy materials manually. Therefore, long term working in building construction industries should be avoided and workers must be given official days off at least two times in a week and two months in a year. The educational programs can be introduced to increase in the workers' knowledge about their health status and work ability. Moreover, based on the present study results workers must be advised to quit the cigarette smoking.

***Study strengths and limitations: ***This is a large sample sized study, the data were collected from six building construction sites, three different ethnicity based population was selected and translation of questionnaire was according to the native language of the workers. A limitation to this is that the findings do not exclude the possible impact of psychosocial job stressors on the musculoskeletal symptoms and due to variety of trades in the construction industry the work specific symptoms were not analyzed.

## References

[1] Meo SA, Al-Drees AM, Al-Masri AA, Al-Rouq F, Azeem MA (2013). Effect of Duration of Exposure to Cement Dust on Respiratory Function of Non-Smoking Cement Mill Workers. Int J Environ Res Public Health.

[2] Pritchard C (2004). Building for Health The construction managers of tomorrow. J R Soc Promot Health.

[3] Tiwary G, Gangopadhyay PK (2011). A review on the occupational health and social security of unorganized workers in the construction industry. Indian J Occup Environ Med.

[4] Forst L, Ahonen E, Zanoni J, Holloway-Beth A, Oschner M, Kimmel L More than training: Community-based participatory research to reduce injuries among Hispanic construction workers. Am J Ind Med.

[5] Piedrahita H (2006). Costs of Work-Related Musculoskeletal Disorders (MSDs) in Developing Countries: Colombia Case. Int J Occup Safety Ergonomics.

[6] Meo SA (2004). Dose responses of years of exposure on lung functions in flour mill workers. J Occup Health.

[7] Meo SA (2006). Lung Function in Pakistani wood workers. 2006. Int J Environ Health Res.

[8] Boschman JS, van der Molen HF, Sluiter JK, Frings-Dresen MH (2012). Musculoskeletal disorders among construction workers: a one-year follow-up study. BMC Musculoskelet Disord.

[9] Kilbom A, Armstrong T, Buckle P (1996). Musculoskeletal disorders: work related risk factors and prevention. Int J Occup Environ Health.

[10] Pope DP, Silman AJ, Cherry NM (2001). Association of occupational physical demands and psychosocial working environment with disabling shoulder pain. Ann Rheum Dis.

[11] Rosecrance JC, Thomas MC, Zimmermann CL (1996). Work-related musculoskeletal symptoms among construction workers in the pipe trades. Work.

[12] Battevi N, Vitelli N (2012). Survey on musculoskeletal disorders in a group of 2755 construction workers in the province of Bergamo. G Ital Med LavErgon.

[13] Bye A (1991). Musculoskeletal disorders among employees in building and construction industry. TidsskrNorLaegeforen.

[14] Hanklang S, Kaewboonchoo O, Silpasuwan P, Mungarndee SS Musculoskeletal Disorders Among Thai Women in Construction-Related Work. Asia Pac J Public Health.

[15] Guo HR, Chang YC, Yeh WY, Chen C W, Guo YL (2004). Prevalence of musculoskeletal disorder among workers in Taiwan: a national study. J Occup Health.

[16] Ashish D, Nimbrate, Fereydoun A, Laura H I, Craig MH (2010). Neck Disorders among Construction Workers: Understanding the Physical Loads on the Cervical Spine during Static Lifting Tasks. Industrial Health.

[17] Gallagher S (2005). Physical limitations and musculoskeletal complaints associated with work in unusual or restricted postures: a literature review. J Safety Res.

[18] Yu-Sheng Yang, David Goldsheyder, Lee-JyyKau (2002). Survey of Musculoskeletal Symptoms among Building Construction Workers in Southern Taiwan. J Occup Therapy Assoc.

[19] Fallentin N (2003). Regulatory action to prevent work-related musculoskeletal disorders – the use of research-based exposure limits. Scand J Work Environ Health.

[20] Marras W, Allread W, Butt D, Fathallah F (2000). Prospective validation of a low-back disorder risk model and assessment of ergonomic intervention associated with manual materials handling tasks. Ergonomics.

